# Polyphenolic Profile and Antioxidant Activity of Leaf Purified Hydroalcoholic Extracts from Seven Mexican *Persea americana* Cultivars

**DOI:** 10.3390/molecules24010173

**Published:** 2019-01-04

**Authors:** Cecilia Castro-López, Israel Bautista-Hernández, María D. González-Hernández, Guillermo C. G. Martínez-Ávila, Romeo Rojas, Adriana Gutiérrez-Díez, Nancy Medina-Herrera, Víctor E. Aguirre-Arzola

**Affiliations:** 1 Laboratory of Chemistry and Biochemistry, School of Agronomy, Autonomous University of Nuevo León, General Escobedo, Nuevo León 66050, Mexico; caslopcec28@hotmail.com (C.C.-L.); bau_israel@hotmail.com (I.B.-H.); romeo.rojasmln@uanl.edu.mx (R.R.); adriana.gutierrezdz@uanl.edu.mx (A.G.-D.); nancy.medinahr@uanl.edu.mx (N.M.-H.); 2 School of Biological Sciences, Autonomous University of Nuevo León, San Nicolás de los Garza, Nuevo León 66450, Mexico; lolis.90.6@gmail.com

**Keywords:** *Persea americana*, antioxidant activity, compound profile, UPLC-ESI-Q/TOF-MS^2^

## Abstract

*Persea americana* (avocado) is a fruit consumed worldwide; however, since avocado leaves are apparently a natural ingredient that can be used as a traditional medicine, they can be a potential source of bioactive compounds. This study aimed to analyze the antioxidant activity of seven Mexican avocado leaf extracts by DPPH^•^, ABTS^•+^, and lipid peroxidation (LPO), and to identify the compound profile by liquid chromatography coupled to mass spectrometry/electron spray ionization. The highest free radical-scavenging activity was observed for Platano Delgado and Criollo 6 avocado cultivars havin IC_50_ values of 271.86 ± 13.69 and 269.56 ± 6.53 for DPPH^•^ and ABTS^•+^ radicals, respectively, while the best result for lipid oxidation inhibition was registered in Criollo 6 cultivar extract. In this study forty-one compounds were detected in avocado leaves of the the seven cultivars analyzed, and of these compounds, eighteen phenolics were identified for first time in such plant material. The present study demonstrated that Mexican cultivars of *Persea americana* possess diverse polyphenolic compounds with strong antioxidant activity, which might be useful in the food and pharmaceutical industries.

## 1. Introduction

*Persea americana* (avocado) is a dicotyledonous tree belonging to the Lauraceae family. It is native of Central America and Mexico, but cultivated in many subtropical and tropical areas (USA, South America, Asia, parts of Europe and Tropical Africa) with high global consumption [1,2]. Avocado is an energetic fruit with great nutritional value and is considered a major tropical fruit, since it is rich in protein and contains fat-soluble vitamins lacking in other fruits, including vitamins A, B, D and E [3].

Different avocado fruit genotypes are cultivated worldwide, and production has expanded considerably in recent years due to rising consumption. The diversity of avocados includes cultivated and wild avocado genotypes with contrasting phenological and morphological characteristics that can create alternative uses to the fresh consumption of the fruit (such as the Mexican avocado, *Persea americana* Mill. var. *drymifolia*) [4]. After its industrial pruning and processing, a large amount of agro-industrial by-products such as seeds, peel, and leaves are generated, which nowadays are discarded with no further applications [5]. However, they could be a rich source of bioactive compounds, that can be applied as functional food ingredients (e.g., vitamins, minerals, proteins, fibers, fatty acids, and polyphenols such as phenolic acids, condensed tannins, and flavonoids, among others) [6,7,8]. These last compounds have been associated with multiple effects (anti-inflammatory, hypo-cholesterolemic, antidiabetic, anticancer, as well as antihypertensive) against some degenerative diseases [9,10].

In this sense, given the variety of uses that are assigned to ethnobotanical genotypes of *P. americana* and residues, several studies have been conducted in order to unveil their biological activity. For example, in America (particularly Latin America), various parts of avocado plants have been used as folk medicine against coughs, bruising, dysentery, diarrhea, wound healing, and hypertension [11]. In addition, the characterization of phenolic components and antioxidant activity of hydroethanolic extracts of avocado skin and seed have demonstrated the predominance of compounds belonging to the group of flavonoids, proanthocyanidins, and hydrocinnamic acids, which have been related with a decrement of the different diseases mentioned above [12,13,14]. Although industrial applications of avocado leaves were not found in the literature, Araújo et al. [15] reported several nutritional and functional properties of avocado by-products including avocado leaf extracts as antimicrobial agents. Then, this information provides potential uses of this plant material in food industry.

Due to the high volume of by-products generated from avocado industries, the possibility of extracting functional biomolecules, and the limited information available in the literature about the phytochemical composition and antioxidant capacity of these residues, especially in leaves, the main focus of this study was to evaluate the antioxidant potential of different extracts from leaves of seven avocado cultivars from the Northeast of Mexico. In addition, extracts were sequentially fractionated using UPLC-ESI-Q/TOF-MS^2^ to identify and characterize their principal chemical constituents.

## 2. Results and Discussion

### 2.1. Antioxidant Capacities from the Different P. americana Cultivars

Due to the fact that the reactions involving antioxidant activity are complex, they should not be judged by a single method [16]. For this reason, in the current study scavenging capacity of free radicals and inhibitory effects on lipid oxidation of seven *P. americana* (leaves) cultivars extracts were evaluated by different methods.

A statistically significant effect (*p* < 0.05) on the antioxidant activities was found (Table 1). In DPPH^•^ assay, the antioxidants are able to reduce the DPPH^•^ radical to the yellow-colored diphenylpicrylhydrazine. As shown in Table 1, the median effective concentration (IC_50_) of the leaf extracts was in the order: PD > Criollo 6 > Campeon > TA = Criollo 1 > Platano > PT, respectively. The lowest IC_50_ values indicated the highest free radical-scavenging activity of the samples. So, the PD cultivar had the highest scavenging activity while the PT cultivar had the lowest, according to the amount or concentration of extracts needed to scavenge 50% of the free radicals. Compared to previous studies, the samples included in our study ranked relatively low, since other authors have reported IC_50_ values between 32.73–164.60 µg·mL^−1^ for avocado leaf and seeds extracts [17,18].

On the other hand, the results of the determination of antioxidant activity by ABTS^•+^ are demonstrated in Table 1. The ranking of the ABTS^•+^ free radical scavenging activity (1/IC_50_, µg·mL^−1^, *p* < 0.05) varied from 269.56 ± 6.52 to 442.72 ± 9.62 µg·mL^−1^ and it was in the following order: Criollo 6 > PD > Campeon > TA > Criollo 1 > Platano > PT. As the IC_50_ concentration and the antioxidant capacity have inversely proportional values, PT was established to have the lowest antioxidant capacity among the seven cultivars tested while PD and Criollo 6 were found to be the richest of all. The IC_50_ values observed for leaves extracts in this study were lower than those reported for peel and comparable with the values registered for seeds in other species of *Persea* [19], which probably makes leaves of *P. americana* a better source for obtaining antioxidative compounds.

Finally, it is known that oxidation of certain fatty acids in biological membranes often lead to the formation and propagation of lipid radicals, uptake of oxygen, rearrangement of the double bonds in unsaturated lipids and even destruction of membrane lipids. For this reason, effects on the inhibition of lipid peroxidation by avocado leaf extracts were tested. The mean concentrations for 50% inhibition (IC_50_) of lipid peroxidation inhibition by *P. americana* cultivar extracts had the following descending order: PD > Platano > Criollo 1 > PT > TA > Campeon > Criollo 6, respectively. As can be seen in Table 1, Campeon and Criollo 6 cultivars showed the lowest IC_50_ values, so they had greater lipid peroxidation inhibition. In agreement with our results, previous studies have shown that the effectiveness of avocado extracts against lipid oxidation is linked to the phenolic contents in peels, seeds and leaves. Rodríguez-Carpena et al. [14] reported that the antioxidant properties exhibited by avocado extracts prevent the lipid oxidation in real food products such as chilled porcine patties. In addition, these avocado extracts from peels and seeds were confirmed as antimicrobial agents against Gram-positive (*Bacillus cereus* and *Listeria monocytogenes*) and Gram-negative (*Escherichia coli*) bacteria. Furthermore, the extracts of avocado seeds and leaves have proven efficacy in the inhibition of the key enzymes linked with Alzheimer’s disease and in the decrement of lipid-induced oxidation [20]. According to the authors, these functional properties are associated with the contents of phenolic compounds, which were higher in leaf extracts compared to seed extracts. Taken together, these results indicate that phenolic compounds identified in avocado leaves could be considered a suitable alternative for food preservation and other pharmacological applications as antioxidants agents.

### 2.2. Phytochemical Constituents Profile of P. americana Cultivars

**Figure 1:**
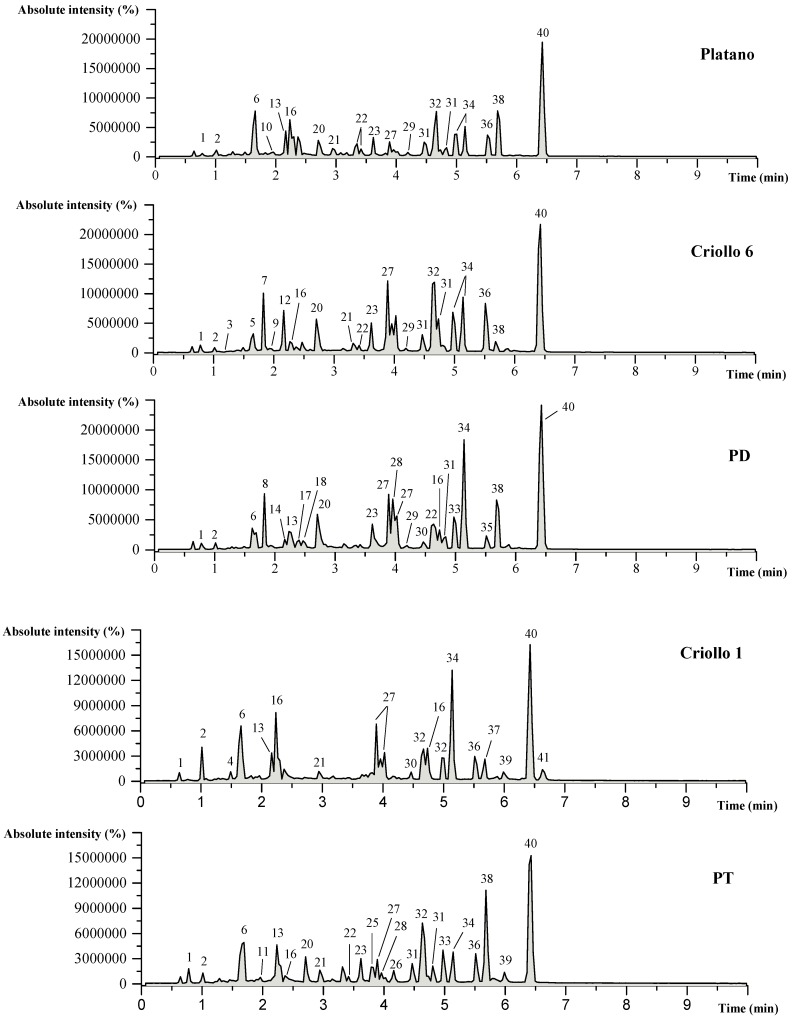

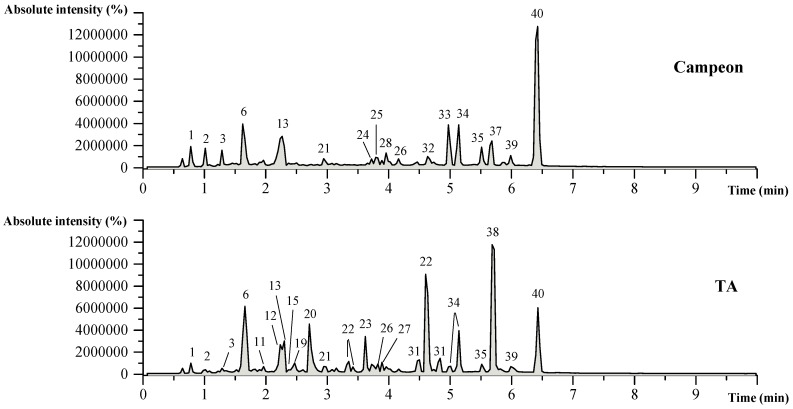
Comparison of UPLC-ESI-Q/TOF-MS^2^ compounds profile of the extracts prepared from *Persea americana* Mill. var. *drymifolia* cultivars. The identification of the peaks is presented in Table 2.

*P. americana* leaf extracts obtained by UAE were analyzed by UPLC-ESI-Q/TOF-MS^2^ in order to characterize the present compounds. A total of thirty-five phenolic compounds and six other polar compounds were found among the seven avocado cultivars (Figure 1). The retention time (RT), molecular ion, fragment ions, molecular formula, and tentative identification are shown in Table 2.

#### 2.2.1. Phenolic Acids

Compound **5** (RT 1.66 min) with [M − H]^−^ at *m*/*z* 209.1988, exhibited a MS^2^ base peak at *m*/*z* 137.2726 and was characterized as 5-hydroxyferulic acid (a precursor of synaptic acid) [21]; while compound **7** (RT 1.83 min) which showed a base peak at *m*/*z* 163.0793 [coumaryl − H]^−^ after loss of glycoside residues was identified as *p*-coumaric acid [22]. Both compounds were present only in the Criollo 6 cultivar. 5-*O*-Caffeoylquinic acid (RT 1.83 min and *m*/*z* 353.1204), an isomer of caffeoylquinic acid, was also found and confirmed by MS^2^ spectra, with a precursor ion at *m*/*z* 191.1986 [23]. This compound was assigned to peak 8 and present only in PD cultivar. Compound **9** (present only in Criollo 6) eluted with a retention time of 1.96 min and *m*/*z* 147.0825 displayed a major fragment at *m*/*z* 103.0221 corresponding to *trans*-cinnamic acid [24]. The observation of a loss of 162 amu (a hexosyl moiety), the base peak at *m*/*z* 163.1347 ([coumaric acid − H]^−^) and the presence of the parent ion at *m*/*z* 325.1297 (coumaroyl hexose) allows us to identify compound **10**, compound present in Platano cultivar, as *p*-coumaroyl hexose [25]. Similarly, peak 18 (PD cultivar) was identified as 3-*O*-*p*-coumaroylquinic acid due to the base peak at *m*/*z* 337.1829, accompanied by a dominant fragment at *m*/*z* 163.1162 corresponding to [*p*-coumaric acid − H]^−^ as previously reported [26]. Roseoside (formate adduct) (peak 19; TA cultivar) was identified according its fragmentation pattern. It showed a precursor ion in negative ionization mode of *m*/*z* 431.2072 [M − H]^−^ and a daughter ion peak at *m*/*z* 385.1421 that suffered the neutral loss of 46 Da [27]. Compound **26** (PT, Campeon, and TA cultivars) presented a pseudomolecular ion [M − H]^−^ at *m*/*z* 551.2521, yielding a MS^2^ fragment at *m*/*z* 443.2231 suggesting that it could be a dihydroxybenzoic acid derivative [28]. Peak 35 in PD, Campeon, and TA cultivars (RT 5.51 min) was easily distinguished by its base peak at *m*/*z* 491.0785, accompanied by a secondary fragment ion at *m*/*z* 328.0980 which present a neutral loss of 163 amu (the loss of hexoside) and the addition of an extra 15 mu (another methyl group to form the main ion at *m*/*z* 328), allowing its identifivcation as dimethyl ellagic acid hexoside according to the fragmentation pattern described by Jia et al. [29]. On the other hand, caffeoyl hexose-deoxyhexoside (peak 37; Criollo 1, and Campeon cultivars) (RT 5.68 min) was assigned based on the parent ion at *m*/*z* 487.1827 and the fragment ion obtained at *m*/*z* 179.2602 due to loss of deoxyhexose plus hexose moieties [37]. Finally, upon fragmentation [M − H]^−^ at *m*/*z* 341.1605 for compound **38** (present in Criollo 1, PT, Campeon, and TA cultivars), a daughter ion at *m*/*z* 179.0185 was produced, which corresponded to caffeic acid moiety, indicating the compound to be a caffeic acid-hexoside as previously described by Chen et al. [37].

As can be seen in Table 2, a total of 11 phenolic acids of two main sub-classes (hydroxycinnamic acids, and hydroxybenzoic acids) were tentatively identified in tested samples. As previously reported in the literature, six compounds such as 5-hydroxyferulic acid (seed), *p*-coumaric acid (pulp and peel), 5-*O*-caffeoylquinic acid (peel), *trans*-cinnamic acid (pulp and seed), 3-*O*-*p*-coumaroylquinic acid (pulp), and a dihydroxybenzoic acid derivative (isomer) (seed) have already been found in different avocado by-products [26,30,36]. It is interesting to highlight the fact that this is the first time that compounds like *p*-coumaroyl hexose, roseoside (formate adduct), dimethyl ellagic acid hexoside, caffeoyl hexose-deoxyhexoside, and caffeic acid-hexoside are reported in *P. americana* samples.

#### 2.2.2. Flavonoids

Compound **4** (present only in Criollo 1) with RT at 1.49 min, was identified as procyanidin trimer A, with *m*/*z* 863.1945 [M − H]^−^. The mayor MS^2^ product ion was *m*/*z* 411.1457 as a result of a RDA fission of the heterocyclic ring system after water elimination, in agreement with the fragmentation reported by Santos et al. [31]. Peak 6 in Platano, PD, Criollo 1, PT, Campeon, and TA cultivars (RT 1.66 min) with a precursor ion at *m*/*z* 451.2266 and main product ion at *m*/*z* 341.1636 was tentatively identified as cinchonain [32]. Compound **11** (PT, and TA cultivars) exhibited the deprotonated molecular ion at *m*/*z* 563.2106, and presented an MS^2^ fragmentation pattern (*m*/*z* 353.1074) typical of asymmetrical di-*C*-glycosides, so it was characterized as apigenin-*C*-hexoside-*C*-pentoside [33]. Taxifolin *O*-pentoside (peak 13; Platano, PD, Criollo 1, PT, and TA cultivars) was tentatively identified by its pseudomolecular ion [M − H]^−^ at *m*/*z* 435.2368, releasing a MS^2^ fragments ion at *m*/*z* 285.1166 characteristic of an [taxifolin − H]^−^ (−132 mu, loss of a pentosyl moiety) [34]. Procyanidin dimer B (which chemical structure is based on the presence of (epi)catechin units, linked by a single bond) presented a pseudomolecular ion at *m*/*z* 577.1094 in RT 2.16 min (PD cultivar). Also, released an MS^2^ spectra ion at *m*/*z* 425.1052 corresponding to an [retro-Diels–Alder reaction (RDA)] unit, characteristic of B-type procyanidins [31]. Compound **15** (present only in TA cultivar) (RT 2.30 min) exhibited a [M − H]^−^ ion at *m*/*z* 431.2063. It suffered the neutral loss of 120 Da to yield the aglycone at *m*/*z* 311.1196. The aglycone was characterized as vitexin (also called apigenin-8-*C*-hexoside) based on the literature data [35]. Peak 16 ([M − H]^−^ at *m*/*z* 433.2227) present in Platano, Criollo 6, PD, Criollo 1, and PT cultivars, was assigned as peonidin 3-*O*-pentoside based on their mass spectra, which showed an MS^2^ signal at *m*/*z* 300.9112 (peonidin; [M − 131]^−^, loss of a pentosyl moiety) [38]. It is worth mentioning that this compound presented two isomers at RT 2.37 and 4.74 min.

Moreover, compound **17** (PD cultivar) presented a pseudomolecular ion [M − H]^−^ at *m*/*z* 289.1178 with a retention time 2.40 min and was identified as catechin. The fragmentation pattern showed fragments at *m*/*z* 245.1362 which can correspond to the loss of the flavonoid A-ring [30]. Quercetin 3,4′-*O*-diglucoside (peak 20; Platano, Criollo 6, PD, PT, and TA cultivars) was found with RT 2.71 min, and experimental [M − H]^−^ at *m*/*z* 625.0995. As previously described by Figueroa et al. [36] in quercetin derivative compounds, occurs a major fragment ion at *m*/*z* 300.1967 in the fragmentation profile, which correspond to an *O*-glycosidic cleavage. On the other hand, pelargonidin 3-*O*-glucoside (with two isomers at RT 2.94 and 3.32 min) in Platano, Criollo 6, Criollo 1, PT, TA, and Campeon cultivars, presented a pseudomolecular ion [M − H]^−^ at *m*/*z* 433.2232. This molecule released a fragment ion at *m*/*z* 271.1117 obtained after loss of 162 mu (hexose moiety) similarly reported in strawberry extracts by Aaby et al. [39]. At retention time 3.35 min and *m*/*z* 609.1123 eluted compound 22 which spectrum of MS^2^ showed a majority fragment at *m*/*z* 301.0183, corresponding to the loss of a quercetin moiety. Because of that, this compound was identified as rutin [26]. Other isomers of rutin were found at retention times 3.42, and 4.63 min (present in Platano, Criollo 6, PD, PT, and TA cultivars). Peak 23 (Platano, Criollo 6, PD, PT, and TA cultivars) was identified as quercetin-*O*-arabinosyl-glucoside. This compound exhibited [M − H]^−^ at *m*/*z* 595.0945 and gave the aglycone at *m*/*z* 300.0086 which is coherent with the derived from a cross-ring cleavage of its sugar moiety (quercetin moiety) [26].

Similarly, another four different quercetin derivatives were detected corresponding to compound **27** (with two isomers at RT 3.89 and 4.02 min) (present in Platano, Criollo 6, PD, Criollo 1, PT, and TA cultivars), compound **28** (PD, PT, and Campeon cultivars), compound **34** (with two isomers at RT 5.01 and 5.14 min) (present in all cultivars), and compound **41** (only in Criollo 1 cultivar). The first one (and the isomer) was identified as quercetin-3-glucoside and displayed an [M − H]^−^ ion at *m*/*z* 463.0943. It suffered the neutral loss of 162 Da showing the aglycone at *m*/*z* 300.0722. The second one was assigned as quercetin glucuronide (RT 3. 96 min and *m*/*z* 477.0708), yielding the major fragment ion at *m*/*z* 301.0646, which corresponds to an *O*-glycosidic cleavage as already mentioned above [44]. The third one with [M − H]^−^ at *m*/*z* 447.1041 gave origin to a fragment ion at *m*/*z* 301.0722 (aglycone) and was assigned to quercetin-*O*-deoxyhesoxide [22]. While the four one was identified as dihydroquercetin-3,5-rhamnoside with *m*/*z* 449.1195 and MS^2^ fragment 303.0182 after elimination of a sugar moiety, which has been previously reported [40]. Peak 29 (RT 4.19 min) in Platano, Criollo 6, and PD cultivars, was tentatively designated as polymethoxylated flavonoid-*O*-pentoside ([M − H]^−^ at *m*/*z* 521.1903) according to the fragmentation pattern yielding a base peak at *m*/*z* 389.1792 with a neutral loss of 132 Da [41]. Cyanidin 3-*O*-arabinoside (peak 30; PD, and Criollo 1 cultivars) with deprotonated molecular ion at *m*/*z* 419.2465 was confirmed based on their mass spectra, which showed an MS^2^ signal at *m*/*z* 287.1895 typical of cyanidin ([M − 132]^−^, loss of a pentosyl moiety) [38]. At retention time 4.47 and 4.80 min (peak 31; Platano, Criollo 6, PD, PT, and TA cultivars) isomers were tentatively identified as luteolin 7-*O*-(2″-*O*-pentosyl) hexoside with experimental *m*/*z* 579.1083. It presented MS^2^ (*m*/*z* 285.2477) fragments indicating the loss of 294 mu (dehydrated glucose moieties). This compound has previously reported by Jeong et al. [42] in pepper samples. Compound **32** with two isomers at RT 4.67 and 4.97 min (Platano, Criollo 6, Criollo 1, PT, and Campeon cultivars) exhibited the deprotonated molecular ion at *m*/*z* 447.1046, and its MS^2^ base peak at *m*/*z* 285.0909 (common characteristic aglycone fragment attributed to kaempferol). Thus, considering bibliographic data it was characterized as kaempferol-*O*-hexoside [43]. Meanwhile, peak 33 in PD, PT, and Campeon cultivars (RT 4.97 min), showed [M − H]^−^ ion at *m*/*z* 461.0767, and loss of glycoside moiety resulting into isorhamnetin aglycone (*m*/*z* 314.1016); thus, was characterized as isorhamnetin-*O*-coumaroyl [22]. Peak **36**, detected at 5.51 min in Platano, Criollo 6, Criollo 1, and PT cultivars, was tentatively assigned to kaempferol-*O*-pentoside according to the molecular formula provided for its accurate mass ([M − 417.1019]^−^). Indeed, the major fragment ion at *m*/*z* 284.0728 revealed a kaempferol moiety, and loss of pentose [43]. Compound **38** (present in Platano, Criollo 6, PD, PT, and TA cultivars) had [M − H]^−^ ion at *m*/*z* 593.1145 with a MS^2^ fragmentation ion at *m*/*z* 284.0763, corresponding to the loss of (epi)catechin monomer, and this fragment revealed two consecutive losses of glucose moieties. So, this compound was tentatively assigned as catechin diglucopyranoside [45]. Finally, kaempferol-*O*-coumaroyl (peak 40; present in all cultivars) (RT 6.43 min) was tentatively identified by the presence of a pseudomolecular ion [M − H]^−^ at *m*/*z* 431.1131 and the release of MS^2^ fragment at *m*/*z* 285.0876, which corresponds to the characteristic aglycone fragment attributed to kaempferol [43].

Concerning to this group of flavonoid compounds, a total of 24 signals (Table 2) present a very distinct profile (flavanols, flavonols, flavones, and anthocyanins) being procyanidin trimer A (peel), procyanidin dimer B (peel), cinchonain (seed and peel), catechin (seed and peel), quercetin 3,4′-*O*-diglucoside (peel), rutin (isomer) (peel), quercetin-*O*-arabinosyl-glucoside (peel), quercetin-3-glucoside (isomer) (peel), luteolin 7-*O*-(2″-*O*-pentosyl) hexoside (isomer) (peel), kaempferol-*O*-hexoside (isomer) (peel), and isorhamnetin-*O*-coumaroyl (peel), the only common compounds found with respect to tested avocado leaves, as previously described by other authors for *P. americana* cultivar [18,36]. However, to the best of our knowledge, apigenin-*C*-hexoside-*C*-pentoside, taxifolin *O*-pentoside (isomer), vitexin, peonidin 3-*O*-pentoside (isomer), pelargonidin 3-*O*-glucoside (isomer), quercetin glucuronide, polymethoxylated flavonoid-*O*-pentoside, cyanidin 3-*O*-arabinoside, quercetin-*O*-deoxyhesoxide (isomer), kaempferol-*O*-pentoside, catechin digluco-pyranoside, kaempferol-*O*-coumaroyl, and dihydroquercetin-3,5-rhamnoside, as far as we are concerned, have never been reported before in avocado plants.

#### 2.2.3. Other Compounds

Due to the nature of avocado fruit, other compounds (six signals) have also been detected in the less hydrophilic part of the chromatographic profile. Compound **1** (present in all avocado cultivars) eluted at RT 0.78 min with *m*/*z* 211.1406, and with a fragment pattern formed at *m*/*z* 101.0183 was identified as perseitol (sugar) in agreement with previous studies on avocado pulp [26]. Another compound (peak 3; present in Criollo 6, Campeon, and TA cultivars) at retention time 1.29 and with *m*/*z* 315.1511 showed a fragmentation ion pattern at *m*/*z* 135.1637 derived from an *O*-glycosidic cleavage and a tyrosol moiety. This evidence suggested that the compound could be identified as hydroxytyrosol glucoside as reported by Tasioula and Tsabolatidou [46] and López-Cobo et al. [26] in olive samples and avocado seeds, respectively.

In addition, four unknown compounds were found with deprotonated molecular ions at *m*/*z* 375.1574, 413.2761, 401.1310, and 551.2495, which displayed MS^2^ spectrum fragments at *m*/*z* 241.1546, 175.1897, 269.0166, and 505.2192 (Peak 2 = all cultivars; Peak 12 = Criollo 6, and TA cultivars; Peak 24 = Campeon cultivar; Peak 25 = PT, and Campeon cultivars) respectively, but their identity could not be established, since to the best of our knowledge, this is the first time that they have been reported in other plant samples.

### 2.3. Possible Genotypic Effect on Antioxidant Activity and Phytochemical Profile

Plants need phenolic compounds for pigmentation, growth, reproduction, resistance to pathogens and for many other functions; the secondary metabolites represent adaptative characters that have been subject to natural selection during evolution. Because the plants cannot escape from their biotic and abiotic stressors, being linked to the ground by means of their root system, and therefore they must stay and protect themselves, the requirement for secondary metabolites have highly, 15–20% of the genes in the plants encode enzymes for secondary metabolites [47]. Moreover, it is well known that antioxidative activity and phytochemical composition of plant extracts depends on plant species, genetic composition, phenological stage, biotic pressures, geographical locations and/or different changes in environmental conditions such as temperature, water availability (drought or precipitation), light intensity, salinity, and pollination, among others [48]. In this study we could relate variations in the antioxidant activity and compounds profile of *P. americana* leaves with differences between genotypes. Most diverse cultivar was TA with 25 detected compounds, while the less diverse was Campeon, with only 16 compounds, including common compounds for all cultivars and those of restricted distribution to only one cultivar. In this respect, Platano Delgado showed four characteristic compounds, followed by Criollo 6 and TA with three compounds of limited distribution; by the other hand, PT did not have any characteristic compound. Campeon and TA were the genotypes with more phenolic acids, reaching four representants in their composition and Platano had only one. In the case of flavonoids, Platano, PD and PD, presented 18 different compounds in their respective composition, while Campeon only presented seven flavonoids. In the case of other compounds, Campeon presented five of the other polar compounds and Platano, PD and Criollo 6 presented only two of them.

There are reports showing that genotype has a significant influence on the profile of phenolic compounds and antioxidant activity in the fruits and leaves of blackberry, raspberry, grape, and olive plants [49,50]. Many phenolics compounds especially flavonoids, are relationated with the plant stress response in nature as: (i) preformed antibiotic compounds (antifungal activity) [51] (ii) saline stress [52]; (iii) water stress [53]; and (iv) other abiotic stress tolerance [54]; nevertheless, the exact role in plant stress response in nature is still under debate [55]. It is still not consensual the existence of differences in chemical markers and antioxidant activity of plant-based products derived from farming systems. It seems that differences are related to other features, as already mentioned above, but the elucidation of the genetic bases (identification of specific genes and ESTs, Expressed Sequence Tags) related to the primary and secondary metabolism of the avocado fruit is needed. Recently, ESTs projects allow to define and characterize the functions of the genes involved in the metabolism and synthesis routes of compounds such as anthocyanins, condensed tannins, and phenolic acids [4]. As above mentioned, these compounds usually are related with protective and adaptative roles, that is why, knowledge of their synthesis and gene expression profiles could help to support integrative agronomic programs and new strategies to use these by-products in future.

## 3. Materials and Methods

### 3.1. Chemicals and Reagents

Gallic acid, linoleic acid, ethanol, 2,2-diphenyl-1-picrylhydrazyl (DPPH^•^), 2,2′-azino-bis (3-ethylbenzthiazoline-6-sulphonic acid) diammonium salt (ABTS^•+^), (±)-6-hydroxy-2,5,7,8-tetramethyl-chromane-2-carboxylic acid (Trolox), and other reagents were purchased from Sigma-Aldrich (Toluca, México). Reagents as sodium carbonate anhydrous (Na_2_CO_3_), sodium hydroxide (NaOH), ferrous chloride (FeCl_2_), potassium phosphate mono-basic (KH_2_PO_4_), potassium phosphate di-basic (K_2_HPO_4_), ferric chloride (FeCl_3_), sodium carbonate (Na_2_CO_3_), potassium persulfate (K_2_S_2_O_8_), and other were of analytical grade; while acetonitrile, methanol, water, and formic acid (CH_2_O_2_) were all LC-MS grade and purchased from Fisher Scientific Chemicals (Fair Lawn, NJ, USA).

### 3.2. Plant Material

Mature leaves from seven Mexican cultivars of avocado (*P. Americana* Mill. var. *drymifolia*), from the Community Bank of Avocado (Latitude 24°6′26.86″ N, Altitude 99°49′42.85″ W, Aramberri, Nuevo León, México), were collected in March 2017. These cultivars were identified as follows: Platano, Criollo 6, Platano Delgado (PD), Criollo 1, Platano Temprano (PT), Campeon, and Todo el Año (TA). The plant material was washed with bi-distilled water and drying in an electric stove at 50 °C for 24 h. Then, an electric mill machine was used to crush the plant material and the particle size was estimated between a range of 2–5 mm. Finally, the powder obtained was stored in plastic bags at room temperature and under conditions of darkness until use.

### 3.3. Extraction of P. americana Polyphenolic Compounds

#### 3.3.1. Ultrasound-Assisted Extraction (UAE)

Ultrasound-assisted extraction was carried out as described by Ariestya-Arlene et al. [56] with some modifications. Each sample (5 g) was extracted with 60 mL of ethanol:water 35:65 (*v*/*v*) at room temperature (as appropriate to maintain a solid: liquid ratio of 1:12 *p*/*v*). All the samples were placed in dark brown-colored reagent bottles with narrow necks and immersed for 40 min at room temperature (25 °C) in an ultrasonic water bath (Model 2510, Sonics and Materials, Branson, MO, USA) at 40 KHz (100% power). Then, the obtained extracts were centrifuged at 3500 rpm (LabNet Hermle Z400k, Labenet International, Edison, NJ, USA) for 15 min at 4 °C to remove solids and stored at −20 °C prior to the analysis.

#### 3.3.2. Purification of the Extracts

From the supernatant obtained, the solvent was removed by evaporation at 50 °C for 48 h, then the dried extract was resuspended in 40 mL of bi-distilled and purified water. The purification was carried out with a stationary phase (Amberlite XAD-16N) and a mobile phase (ethanol) for recovering only the compounds of interest. Namely, 20 mL of sample were added to the chromatographic column, washed with bi-distilled water and subsequently the polyphenolic compounds were recovered with ethanol. The solvent of the purified extract was removed by evaporation at 50 °C for 48 h.

### 3.4. Antioxidant Activities Evaluation

Purified extract (prepared according with the previous section) was re-dissolved in water, to a final concentration of 2 mg·mL^−1^ for antioxidant activities evaluation. The final solutions were further diluted to 7 different concentrations to be submitted to distinct antioxidant evaluation in vitro assays. All the results were expressed in IC_50_ values (sample concentration providing 50% of antioxidant activity or 0.5 of absorbance in the reducing power assay) which was determined from the graph of the free radical scavenging activity (%) against the extract concentration.

#### 3.4.1. DPPH^•^ Radical Scavenging Activity

The antioxidant activity in the extracts was evaluated as the DPPH^•^ free radical-scavenging activity. This activity was estimated using the method of Brand-Williams et al. [57] with minor modifications. Briefly, 50 µL of each extract was added to a 2950 µL DPPH^•^ methanol solution (60 µM). The reaction mixture was vortexed thoroughly and left in the dark at room temperature for 30 min. The absorbance of the mixture was measured spectrophotometrically at 517 nm. Gallic acid was used as reference. The ability to scavenge the radical was calculated by the following Equation (1):

Inhibition (%) = [(A control − A sample)/A control] × 100(1)

where A control is the absorbance of the control reaction (containing all reagents except the test compound) and A sample is the absorbance with the test compound.

#### 3.4.2. ABTS^•+^ Radical Scavenging Activity

The inhibition assay of the ABTS^•+^ radical was conducted according to the methodology proposed by Van den Berg et al. [58] with minor modifications. The ABTS^•+^ radical cation was generated by mixing an ABTS^•+^ (7 mM) aqueous solution with potassium persulfate (2.45 mM) in dark at room temperature for 12 h before use. Diluted solutions of ABTS^•+^ were prepared in ethanol until a value of 0.700 ± 0.002 nm absorbance was obtained. Varying concentrations of the plant extracts (50 μL) was allowed to react with 950 μL of the ABTS^•+^ solution and after 1 min of reaction the absorbance was measured at a wavelength of 734 nm. The capacity to inhibit the radical (expressed as percent inhibition of the ABTS^•+^ radical) was compared with Trolox as a standard and calculated according to the following Equation (2):

Inhibition (%) = [(A control − A sample)/A control] × 100(2)

where A control is the absorbance of the control reaction (containing all reagents except the test compound) and A sample is the absorbance with the test compound.

#### 3.4.3. Linoleic Acid Peroxidation Inhibition Assay

This test system was developed to determine the ability of substances to inhibit the generation of hydroxy peroxides at the early stages of the oxidation of linoleic acid and, this oxidation is monitored by measuring the values of conjugated dienes spectrophotometrically. The assay was determined as described by Zou et al. [59] with a sight modification. First, the linoleic acid solution was prepared by mixing 0.6 g of linoleic acid and 1.5 g of Tween 20 in 8 mL of ethanol. Then, the plant extract (50 µL) was mixed with linoleic acid solution (100 µL) and acetate buffer (1500 µL, 0.02 M, pH 4). Controls contained 50 µL of distilled water. The samples were homogenized in vortex and incubated at 37 °C for 1 min. Once achieved 1 min, 750 µL of 50 M FeCl_2_ solution (0.01 g FeCl_2_ and 0.017 g EDTA diluted to 100 mL with distilled water) were added to induce the lipid oxidation and incubated for 24 h at 37 °C. Two aliquots (250 µL) were withdrawn during this period, at 0 and 24 h. Each aliquot was processing in the moment as following: the aliquot was added to NaOH solution (1 mL, 0.1 M, in ethanol at 10%, *v*/*v*) to stop the oxidation process; after ethanol (2.5 mL, 10%, *v*/*v*) was placed to dilute the sample. Then, the absorbance of the samples was measured at 232 nm. Ethanol (10%, *v*/*v*) was used as blank. Percent inhibition of linoleic acid oxidation was calculated with the following Equation (3):

Lipid oxidation inhibition (%) = [(A − B)/A] × 100(3)

where A is the difference between the absorbance of distilled water (as control) after 24 h and 0 h of incubation, and B is the difference between the absorbance of each extract sample after 24 h and 0 h of incubation.

### 3.5. UPLC-ESI-Q/TOF-MS^2^ Analysis for Compounds

The seven samples were analyzed using an Acquity ultra-performance liquid chromatography (UPLC) system (Waters, Milford, MA, USA) consisting of an auto-sampler and a binary pump equipped with a 10 µL Loop (partial Loop injection mode). A Waters BEH PHENYL (2.1 mm × 100 mm, 1.7 µm; WATERS, Waxford, Ireland) analytical column thermostatted at 40 °C was used. The chromatographic separation was conducted according to the methodology proposed by Castro-López et al. [60]. The solvents used were: (A) 0.1% (*v*/*v*) formic acid in water, and (B) 100% acetonitrile with a constant flow rate of 0.3 mL·min^−1^. The elution gradient established (for 10 min) was 97% A for 1.10 min, followed by multiple gradients from 5% B to 15% B from 1.10 to 4.40 min, holding 15% B for 4.60 min, getting back to the initial conditions (3% B) in 1 min and re-equilibration of the column, using an injection of 3 µL of the sample. MS detection was performed in a quadrupole-time-of-flight (Q-TOF™, Waters, Milford, MA, USA) orthogonal accelerated Q-TOF mass spectrometer, equipped with an electrospray ionization source (ESI). The full screen mass spectra detection was carried out in the negative ion mode in a mass range from 50–1200 *m*/*z* and using a capillary voltage of 3.0 kV, a dry gas temperature of 210 °C, a dry gas flow of 8.0 L·min^−1^, a nebulizer pressure of 2.0 bar, and spectra rate of 1 Hz. Moreover, automatic MS^2^ experiments were performed using a ramp collision energy of 15–35 V with argon as collision gas and adjusting the scan time every 1 s. The identification of the phenolic compounds in the various extracts was obtained by using the full mass spectrum and its unique mass fragmentation spectrum. Comparison of the observed MS^2^ spectra with those found in the literature and Databases was the main tool for identification of the compounds.

### 3.6. Experimental Design and Statistical Analysis

All experiments were conducted at three levels of measurements and results reported as mean ± standard deviation (SD). The data were analyzed by using one-way analysis of variance (ANOVA) followed by Tukey’s HSD Test with *p* = 0.05. Statistical analysis was performed using Minitab 17 Statistical Software (Minitab, Inc., State College, Pennsylvania, PA, USA).

## 4. Conclusions

The present study demonstrated that avocado leaves represent an important source for the recovery of added-value compounds with high antioxidant activity, which have potential applications as bioactive antioxidant agents for the treatment of several diseases and in the development of novel food products. The phenolic analysis of this plant material allowed the identification of at least thirty-seven bioactive compounds (phenolic acids and flavonoids mainly), which may have application in the medicine field. Phytochemical diversity and variation are evident when cultivars are compared, which could be the result of the genetic constitution as they were grown under same climatic conditions and samples were taken from mature and productive avocado trees. Nevertheless, despite these positive results more detailed studies are needed to confirm this correlation with genotypes, increasing the number of individuals, detecting more phytochemical diversity and complementing with gene expression studies.

## Figures and Tables

**Table 1:** Comparison of antioxidant activity (DPPH^•^, ABTS^•+,^ and LPO) of leaf extracts of different *Persea americana* Mill. var. *drymifolia* cultivars.

Cultivar	Antioxidant Activity (IC_50_ Value µg·mL^−1^)		
	DPPH^•^	ABTS^•+^	LPO
Platano	1514.24 (±20.02) ^b^	416.20 (±11.65) ^b^	1708.18 (±12.39) ^b^
Criollo 6	308.43 (±27.67) ^e^	269.56 (±6.52) ^f^	810.34 (±7.63) ^g^
PD	271.86 (±13.69) ^f^	287.52 (±6.63) ^e^	1829.60 (±14.01) ^a^
Criollo 1	1029.71 (±6.33) ^c^	415.21 (±1.61) ^b^	1255.37 (±4.16) ^c^
PT	1618.71 (±23.96) ^a^	442.72 (±9.62) ^a^	1229.66 (±13.12) ^d^
Campeon	984.97 (±5.34) ^d^	318.32 (±19.62) ^d^	837.17 (±6.53) ^f^
TA	1026.89 (±7.88) ^c^	388.95 (±8.70) ^c^	1048.88 (±28.30) ^e^

IC_50_ values correspond to the sample concentration achieving 50% of antioxidant activity or 0.5 of absorbance in reducing power assay. Gallic acid equivalents for DPPH^●^, Trolox equivalents for ABTS^●+^, and Linoleic acid inhibition for LPO. In each column, different letters mean significant (*p* < 0.05) differences between the cultivars samples. Data with additional letters in superscript were statistically different (*p* < 0.05).

**Table 2:** Mass spectral data and tentative identification of present compounds in *Persea americana* Mill. var. *drymifolia* cultivars (leaves). For peak assignation see Figure 1.

Peak N°	Rt (min)	*m*/*z* Experimental [M − H]^−^	*m*/*z* Calculated [M − H]^−^	Tentative Assignment	Molecular Formula	Error (ppm)	MS^2^ Fragment Ion	Cultivar							Reference
								Platano	Criollo 6	PD	Criollo 1	PT	Campeon	TA	
Phenolic acids															
5	1.66	209.1988	209.0533	5-Hydroxyferulic acid	C_10_H_10_O_5_	695.99	137.2726		x						[20]
7	1.83	163.0793	163.0478	*p*-Coumaric acid	C_9_H_8_O_3_	193.19	163.0441		x						[21]
8	1.83	353.1204	353.0956	5-*O*-Caffeoylquinic acid	C_16_H_18_O_9_	70.23	191.1986			x					[22]
9	1.96	147.0825	147.0529	*trans*-Cinnamic acid	C_9_H_8_O_2_	201.28	103.0221		x						[23]
10	1.96	325.1297	325.0928	*p*-Coumaroyl hexose	C_15_H_17_O_8_	113.50	163.1347	x							[24]
18	2.47	337.1829	337.1007	3-*O*-*p*-Coumaroylquinic acid	C_16_H_18_O_8_	243.84	163.1162			x					[25]
19	2.47	431.2072	431.1922	Roseoside (formate adduct)	C_20_H_31_O_10_	34.78	385.1421							x	[26]
26	3.82/4.16	551.2521	551.2497	Dihydroxybenzoic acid derivative	C_28_H_39_O_11_	4.35	443.2231					x	x	x	[27]
35	5.51	491.0785	491.1065	Dimethyl ellagic acid hexoside	C_22_H_22_O_13_	−57.01	328.0980			x			x	x	[28]
37	5.68	487.1827	487.1821	Caffeoyl hexose-deoxyhexoside	C_22_H_31_O_12_	1.23	179.2602				x		x		[29]
39	5.99	341.1605	341.0956	Caffeic acid-hexoside	C_15_H_18_O_9_	190.26	179.0185				x	x	x	x	[29]
Flavonoids															
4	1.49	863.1945	863.190713	Procyanidin trimer A	C_45_H_36_O_18_	4.38	411.1457				x				[30]
6	1.66	451.2266	451.1112	Cinchonain	C_24_H_20_O_9_	255.81	341.1636	x		x	x	x	x	x	[31]
11	1.96	563.2106	563.1484	Apigenin-*C*-hexoside-*C*-pentoside	C_26_H_28_O_14_	110.45	353.1074					x		x	[32]
13	2.16/2.23	435.2368	435.1011	Taxifolin *O*-pentoside	C_20_H_20_O_11_	311.88	285.1166	x		x	x	x		x	[33]
14	2.16	577.1094	577.1351	Procyanidin dimer B	C_30_H_25_O_12_	−44.53	425.1052			x					[30]
15	2.30	431.2063	431.1061	Vitexin	C_21_H_20_O_10_	232.42	311.1196							x	[34]
16	2.37/4.74	433.2227	433.1089	Peonidin 3-*O*-pentoside	C_21_H_21_O_11_	262.75	300.9112	x	x	x	x	x			[35]
17	2.40	289.1178	289.0717	Catechin	C_15_H_13_O_6_	159.47	245.1362			x					[36]
20	2.71	625.0995	625.1488	Quercetin 3,4′-*O*-diglucoside	C_27_H_30_O_17_	−78.86	300.1967	x	x	x		x		x	[37]
21	2.94/3.32	433.2232	433.1140	Pelargonidin 3-*O*-glucoside	C_21_H_21_O_10_	252.12	271.1117	x	x		x	x	x	x	[38]
22	3.35/3.42/4.63	609.1123	609.1539	Rutin	C_27_H_30_O_16_	−68.29	301.0183	x	x	x		x		x	[25]
23	3.62	595.0945	595.1382	Quercetin-*O*-arabinosyl-glucoside	C_26_H_28_O_16_	−73.42	300.0086	x	x	x		x		x	[25]
27	3.89/4.02	463.0943	463.0960	Quercetin-3-glucoside	C_21_H_20_O_12_	−3.67	300.0722	x	x	x	x	x		x	[39]
28	3.96	477.0708	477.0752	Quercetin glucuronide	C_21_H_18_O_13_	−9.22	301.0646			x		x	x		[39]
29	4.19	521.1903	521.1875	Polymethoxylated flavonoid-*O*-pentoside	C22H33O_14_	5.37	389.1792	x	x	x					[40]
30	4.47	419.2465	419.0983	Cyanidin 3-*O*-arabinoside	C20H19O_10_	353.61	287.1895			x	x				[33]
31	4.47/4.80	579.1083	579.1433	Luteolin 7-*O*-(2″-*O*-pentosyl) hexoside	C26H28O_15_	−60.43	285.2477	x	x	x		x		x	[41]
32	4.67/4.97	447.1046	447.0932	Kaempferol-*O*-hexoside	C21H19O_11_	25.49	285.0909	x	x		x	x	x		[42]
33	4.97	461.0767	461.1167	Isorhamnetin-*O*-coumaroyl	C22H22O_11_	−86.74	314.1016			x		x	x		[21]
34	5.01/5.14	447.1041	447.1011	Quercetin-*O*-deoxyhesoxide	C21H20O_11_	6.70	301.0722	x	x	x	x	x	x	x	[21]
36	5.51	417.1019	417.0905	Kaempferol-*O*-pentoside	C20H18O_10_	27.33	284.0728	x	x		x	x			[42]
38	5.68	593.1145	593.1511	Catechin diglucopyranoside	C27H29O_15_	−61.70	284.0763	x	x	x		x		x	[43]
40	6.43	431.1131	431.0983	Kaempferol-*O*-coumaroyl	C21H19O_10_	34.16	285.0876	x	x	x	x	x	x	x	[42]
41	6.63	449.1195	449.1167	Dihydroquercetin-3,5-rhamnoside	C21H22O_11_	6.23	303.0182				x				[44]
Other compounds															
1	0.78	211.1406	211.0901	Perseitol	C_7_H_16_O_7_	239.23	101.0183	x	x	x	x	x	x	x	[25]
2	1.01	375.1574	375.1508	Unknown	C_13_H_27_O_12_	17.59	241.1546	x	x	x	x	x	x	x	-------
3	1.29	315.1134	315.1163	Hydroxytyrosol glucoside	C_14_H_20_O_8_	−9.20	135.1637		x				x	x	[25,45]
12	2.16	413.2761	413.2756	Unknown	C_19_H_41_O_9_	1.20	175.1897		x					x	-------
24	3.72	401.1310	401.1300	Unknown	C_14_H_25_O_13_	2.49	269.0166						x		-------
25	3.79	551.2495	551.2497	Unknown	C_28_H_39_O_11_	−0.54	505.2192					x	x		-------

x = indicates presence in cultivars. --- = not reference.
